# Dysbiotic Gut Microbiota Modulation by Aronia Fruits Extract Administration

**DOI:** 10.3390/life13010032

**Published:** 2022-12-22

**Authors:** Emanuel Vamanu, Florentina Gatea, Ionela Avram, Gabriel Lucian Radu, Sandeep Kumar Singh

**Affiliations:** 1 Faculty of Biotechnology, University of Agricultural Sciences and Veterinary Medicine, 011464 Bucharest, Romania; 2 Centre of Bioanalysis, National Institute for Biological Sciences, 296 Spl. Independentei, 060031 Bucharest, Romania; 3 Department of Genetics, University of Bucharest, 36-46 Bd. M. Kogalniceanu, 5th District, 050107 Bucharest, Romania; 4 Indian Scientific Education and Technology Foundation, Lucknow 226002, India

**Keywords:** bioavailability, pattern, phenolic compounds, in vitro, antioxidant

## Abstract

The administration of chokeberry extract in vitro in the GIS1 system was evaluated for the modulation capacity of the dysbiotic pattern resulting from the consumption of stevia. The microbial pattern determined by molecular method, the metabolomic one (fatty acids), the evolution of the antioxidant status, and the cytotoxic effect were determined comparatively for six months. This study presented for the first time that Aronia extract has a strong antimicrobial effect but also a presence of new organic acids that can be used as a biomarker. The functional supplement had the impact of a gradual increase in antioxidant status (DPPH scavenging activity) for up to three months and a subsequent decrease correlated with the reduction of the microbial load (especially for Enterobacteriaceae). The effect on metabolomic activity was specific, with butyric acid being generally unaffected (0.6–0.8 mg/mL) by the antimicrobial effect manifested after three months of administration. The pH was strongly acidic, corresponding to the constant presence of maximum values for acetic and lactic acid. The non-selective elimination of a part of the microbiota could also be correlated with a decrease in metabolomic efficiency. The results in the GIS1 system indicated for the first time that the controlled use of this extract had a pronounced antimicrobial and cytotoxic effect. This has helped to correct the dysbiotic pattern that results after the long-term use of sweeteners based on an increase of 0.2 log UFC/mL for favorable strains.

## 1. Introduction

Gut microbiota modulation represents an accepted strategy for controlling and alleviating some pathologies with a high incidence, such as type 2 diabetes or even the prevention of colorectal cancer [1]. Current data reveal normalization of the microbial pattern after the administration of functional supplements. Many of these data are obtained in vitro and are supported by specific biomarkers (short-chain fatty acids—SCFAs) and the increase in antioxidant potential [2]. Modulation using functional products (probiotics, phenolic compounds with prebiotic and/or antimicrobial action, etc.) is a new alternative that responds to current requirements regarding the fight against chronic pathologies associated with intestinal dysbiosis [3]. The consumption of *Aronia melanocarpa* in the form of pasteurized juice or the administration of atomized extract is a new approach in Romania. The number of producers has increased in the last decade along with the awareness of the role that the content of polyphenolic compounds has on human health [4]. The anti-inflammatory effect is one demonstrated by in vitro studies, which makes Aronia products a helpful candidate in managing dysbiosis associated with chronic pathologies [5]. The association between the dysbiotic state and the presence of an inflammatory process represents an explanation demonstrated in vitro. The action of supporting the antioxidant potential through the consumption of Aronia determines the use of the fruit in the control of dysfunctions associated with the dysbiotic states of the microbiota of some target groups and in the modulation of essential metabolomic biomarkers that can improve critical physiological processes [6].

The initiation of in vitro studies represents the fastest alternative to confirm the prebiotic action of the functional compounds of Aronia in chronic pathologies such as type 2 diabetes or obesity [7]. As a therapeutic strategy, the antioxidant and anti-inflammatory properties of Aronia as well as the content of catechins, determine modulation processes at the level of the colon microbiota and diminish the effect of type 2 diabetes but also of other associated chronic diseases [8]. Thus, the present study aimed to characterize and extract from *Aronia melanocarpa* fruits and demonstrate the modulation of the dysbiotic microbiota of children after consuming food sweeteners. The in vitro study, carried out in the GIS1 simulator, followed the effect of the extract on the antioxidant status as a strategy to reduce inflammatory progression but also to establishm the metabolomic pattern through CZE and the evolution of the microbial profile through qPCR analysis.

## 2. Materials and Methods

### 2.1. Obtaining the Functional Extract

*Aronia melanocarpa* fruits were cultivated in an ecological culture in the Ionaseni village, Vârfu Câmpului county, Botoșani, Romania. After harvesting, all fruits were washed two times and dried then placed in borosilicate glasses of 500 mL capacity (20 g fruits/100 mL solvent) and mixed with ethanol:water: acetic acid (50:49.5:0.5) [9]. The extraction took place at room temperature for a maximum of 48 h. The extract was filtered under vacuum with Whattman no. 1 filter paper. The solvent from the remaining solution was evaporated in a Buchi rotary evaporator, and the extract was lyophilized in a Martin Christ freeze drier.

### 2.2. Microbiota Restoration and In Vitro Simulations

Microbiota from the ColHumB collection from the Faculty of Biotechnology in Bucharest, Romania was used. A minimum number of three dysbiotic microbiota was used, obtained by in vitro simulation following the regular consumption of stevia (as a sweetener) over at least one year. The dysbiotic pattern was preserved in glycerol at −80 °C and was revitalized by culturing in peptone water for 7–10 days [6].

The studies were carried out in the in vitro system GIS1 (www.gissystems.ro) over six months with minor changes compared to the previous study [10]. An administration equivalent to 500 mg dried extract/day, a type 00 gastro-resistant capsule for prolonged release, and a minimal loss of interaction with the recovered dysbiotic microbiota was considered. The average pH at which the studies were carried out was maintained at 6.8, the inoculation rate being 10% recovered dysbiotic microbiota. After the sterile collection of the samples, they were centrifuged, and the microbial load was preserved in 20% glycerol at −15 °C. After obtaining the samples, a similar volume of fresh sterile medium was added using a peristaltic pump.

### 2.3. Analysis of Antioxidant Status In Vitro (DPPH Scavenging Assay)

DPPH scavenging activity was determined by a spectrophotometric method [11]. The reaction mixture was obtained by mixing DPPH 0.1 mM with an equal proportion of the sample. After 20 min, the absorbance was read at 517 nm. The control was the value obtained after the microbiota revitalization.

### 2.4. Determination of the Level of Total Phenolic Compounds

Total phenolic content was determined by a spectrophotometric method using the Folin–Ciocalteu reagent [11]. The reaction mixture was 1 mL sample, 1 mL ethanol, 5 mL distilled water, 0.5 mL Folin–Ciocalteu reagent (1/2 with distilled water), and 1 mL Na_2_CO_3_ 5%. The mixture was kept in the dark for a maximum of one hour, and the absorbance was read at 725 nm. The control was the value obtained after the microbiota revitalization.

### 2.5. Organic Acids Determination

Separation and quantification of organic acids were possible using a zonal capillary electrophoresis technique. The separations were performed on an Agilent 7100 capillary electrophoresis instrument (Agilent Technologies, Ratingen, Germany). The diameter of the silica capillary was 50 cm, and the effective length was 63 cm. The parameters of the method were previously optimized [12]. In short, the background electrolyte (BGE) contained 0.5 M H3PO4, 0.5 mM CTAB (cetyltrimethylammonium bromide) (pH adjusted with NaOH to 6.24), and 15 % methanol. BGE was filtered on 0.2 μm membranes (Millipore, Bedford, MA, USA) and degassed before use. The applied voltage was −20 kV, and the UV detection was performed at 200 nm. Sample injection was performed using the hydrodynamic mode, 35 mbar/10 s, while the capillary was maintained at a constant temperature of 25 °C. The capillary was flushed between runs with 0.1 M NaOH for 2 min, H_2_O for 2 min, and the background electrolyte for 2 min. The samples representing small volumes of the culture medium from the in vitro system GIS1 were centrifuged (5000 rpm), filtered on 0.2 μm membranes (Millipore, Bedford, MA, USA), and then separated by CZE (Figure S1). The elution order of the standards was formic acid, oxalic acid, succinic acid, acetic acid, propionic acid, lactic acid, butiric acid, benzoic acid, isovaleric acid, phenyllactic acid, and hydroxyphenyllactic acid. The identification of the SCFAs peaks was achieved by comparing the retention times and by standard addition. The calibration curves made for each standard showed good linearity (r^2^ > 0.995) for the concentration range tested. The detection limits (LODs) varied between 0.001–1.45 µg mL^−1^ and the quantification limits (LOQs) between 0.004–4.35 µg mL^−1^.

### 2.6. Chromatographic Analysis of Polyphenolic Compounds

Analyses of polyphenolic compounds were performed on a Shimadzu 20AD high-performance liquid chromatography system equipped with a Shimadzu SPD-M20A Diode Array Detector (HPLC-DAD), a thermostated column compartment (CTO-20AC), autosampler (SIL-20A HT), pumps (LC-20AD), and degasser (DGU-20A3). The separation was carried out on a Kinetex C18 chromatographic column with particle diameters 5 μm, 150 × 4.6 mm. Data were analyzed using LCsolution software. The separation method used a mobile phase consisting of (A) distilled water brought to pH = 2.5 with phosphoric acid and (B) acetonitrile, phase gradient elution: 30% to 50% B in 0.00–28.00 min followed by 50% to 62% B in 28.00–45.00 min then 62% to 70% B in 45.00–50.00. The mobile phase flow was 0.8 mL/min, the injection volume 10 µL, and the chromatographic column was maintained at a temperature of 35 °C. The detection and quantification of the analytes of interest were carried out at the wavelengths 280 nm, 320 nm, and 360 nm [13]. The samples subjected to separation by HPLC–DAD were prepared similarly to those from electrophoretic separation. The identification of the phenolic compounds was achieved by comparing their retention times with those of the standards. The elutions order of the standards was gallic acid, caftaric acid, catechin, chlorogenic acid, caffeic acid, epicatechin, cynarin, coumaric acid, rutin, ellagic acid, ferulic acid, isoquercitin, hesperidin, rozmarinic acid, myrecitin, daidzein, quercetin, cinnamic acid, apigenin, naringenin, genistein, kaemferol, rhamnetin, chrysin, pinocembrin, and pinostrobin (Figures S2–S4). The calibration curves (r^2^ > 0.995) were made for each standard, and LODs (between 0.16–2.52 µg mL^−1^) and LOQs (between 0.48–7.56 µg mL^−1^) were calculated.

### 2.7. Analysis of Microbiota Pattern Evolution

One mL of each sample was used for DNA extraction by applying the QIAamp DNA Stool Mini Kit (QIAGEN, Hilden, Germany). The DNA concentration and purity were determined by reading the absorbance at NanoDrop 8000 spectrophotometers (Thermo Fisher Scientific, Waltham, MA, USA). The primers’ coverages were analyzed in the Arb-SILVA database (https://www.arb-silva.de/ accessed on 30 June 2022). An amount of 5 ng of DNA was introduced in each reaction. The other PCR amplification conditions, including primer details, were presented in a previous study [14].

### 2.8. Determination of the Cytotoxic Effect

The cytotoxic effect of extracts was assessed by measuring HT-29 cell viability using Vybrant^®^ MTT Cell Proliferation Assay Kit (Thermo Fisher Scientific, Waltham, MA, USA). HCT-8 cells were cultivated in RPMI 1640 (Lonza, Basel, Switzerland) supplied with 10% FBS (Biochrom, Berlin, Germany) in polystyrene 96 well plates at 37 °C, 5% CO_2_ until they reached a 75% confluence, passage 43. The media was removed, and the cells were incubated for 24 h in fresh media and extracts in two different concentrations: 10% and 1%. After the incubation, the medium was removed, and the cells were washed one time with warm PBS and incubated with MTT solution for 2.5 h. The dye was solubilized with DMSO, and the plate was read at 540 nm using Synergy HTX (Biotek, Winooski, VT, USA). Cell viability was calculated using the formula: % survival = (mean experimental absorbance/mean control absorbance) × 100. Ethanol 50% was used as a control.

All the parameters were evaluated in triplicate, and the results were expressed as the mean and standard deviation (SD) values of three independent determinations. The statistical analysis and means/SD values were calculated using GraphPad Prism^®^ version 9.1.0 (GraphPad Software, San Diego, CA, USA). For DPPH radical scavenging activity, the one-way ANOVA was used followed by a Brown–Forsythe test while for the antiproliferative assay, we applied a two-way ANOVA test. The significance level for the calculations was *p* express.

## 3. Results

### 3.1. The Evolution of the Antioxidant Status and the Amount of Total and Individual Phenols In Vitro

**Figure 1:**
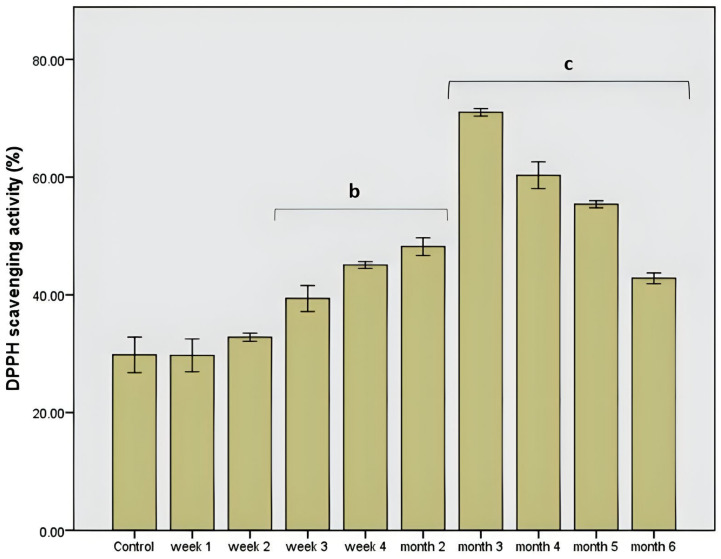
DPPH scavenging activity after chokeberry extract administration in vitro. Different letters represent significant statistical differences (*p* ≤ 0.05) between samples/samples vs. control, n = 3.

As shown in Figure 1, the administration of the extract resulted in a rapid and stable increase in protection against free radicals. The dysbiotic pattern showed an approximately 30% lower antioxidant level than the rest of the intervals when chokeberry extract was administered. After the sharp decrease in the second week correlated with a general antimicrobial effect on the microbiota, the increase in antioxidant protection was gradual until the third month with a value of about 70%, *p* = 0.001. Then a decrease in this in vitro value was observed, an effect that was also accompanied by a normalization of the microbial pattern and a moderate variation of the pH value of the in vitro simulation environment.

**Figure 2:**
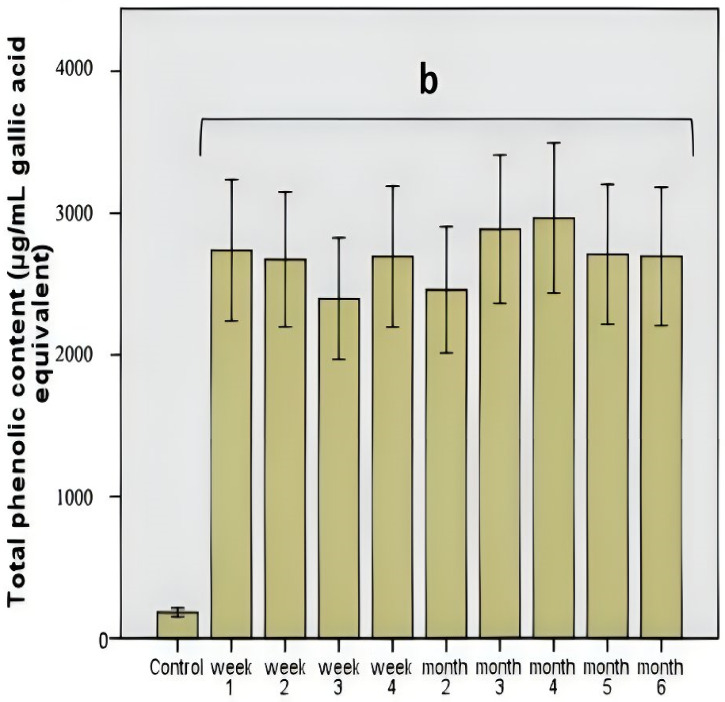
Total phenolic content after chokeberry extract administration in vitro. Different letters represent significant statistical differences (*p* ≤ 0.05) between samples/samples vs. control, n = 3.

The total polyphenolic level was constant, which demonstrated the stability of this polyphenolic pattern compared to other products, such as the influence of some functional foods (mushrooms) [15]. The manifested stability was correlated with a strong antimicrobial effect influencing the use of these compounds as a carbon source or with a microbial modulation process based on the antimicrobial effect. The determined values did not show significant variations (*p* = 0.001; Figure 2), which demonstrated a correlation between the total polyphenolic content and the average value of DPPH scavenging activity (46.8%, *p* = 0.01).

The chromatographic analysis of the samples allowed the identification of individual polyphenolic compounds. They were identified: epicatechin, ellagic acid, cinnamic acid, apigenin, chlorogenic acid, cafeic acid, coumaric acid, naringenin, ferulic acid, rutin, isoquercetin, and kaempferol (Figures S2–S4). These individual compounds, together with other compounds such as anthocyanins, are included in the composition of the chokeberry extract [16].

### 3.2. Cytotoxic Effects of Aronia Extract

**Figure 3:**
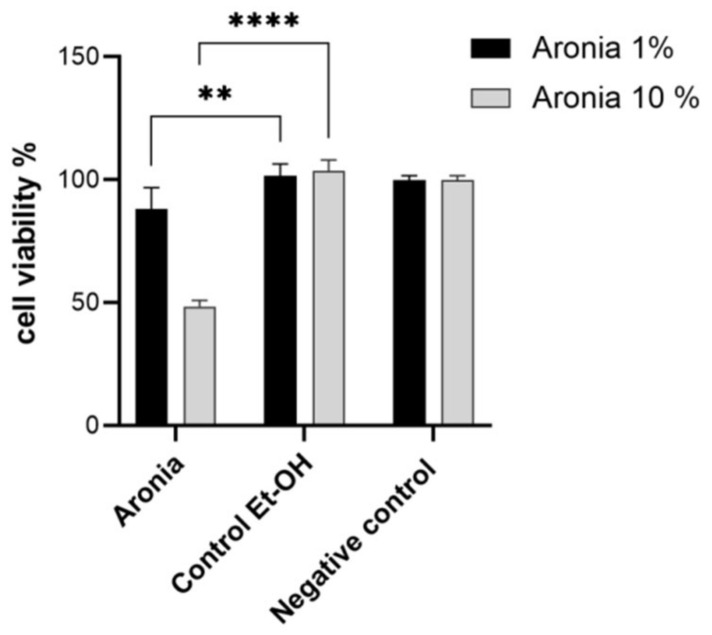
Effect of chokeberry extract on cell viability at concentrations of 1 and 10% compared to controls. Different asterisks represent significant statistical differences (*p* ≤ 0.05) between samples vs. control, n = 3.

In Figure 3, the evaluation of the cytotoxic effect showed significant changes compared to controls (*p* ≤ 0.01 compared to 50% ethanol as control) at the concentration of 1% extract. A concentration of 10% provided a significant cytotoxic effect, which was approximately 40% more pronounced (*p* ≤ 0.0001 vs. 50% ethanol as control). These results demonstrated a necessary increase in cytotoxicity against the proliferation of possible conditions favoring the occurrence of colon cancer. The results were correlated with an antioxidant effect in vitro (Figure 1), which was more pronounced with an increasing administration period.

### 3.3. The Effect of Modulation of the Dysbiotic Microbiota In Vitro

**Figure 4:**
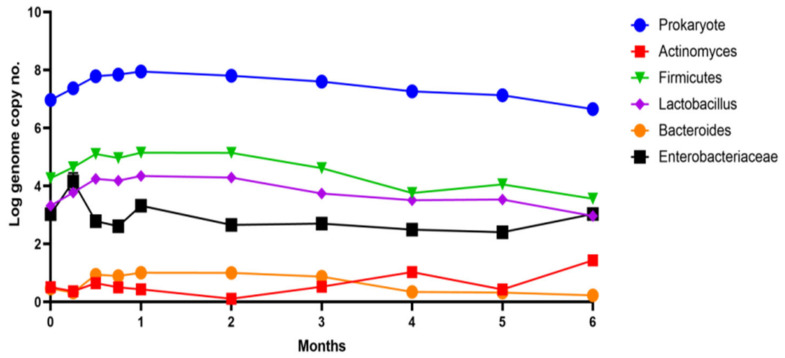
Microbiota pattern after six months of administration of chokeberry extract in vitro.

A rapid correction was determined from the simulation’s first month (Figure 4) until the middle of the second month. Subsequently, the entire determined pattern showed reductions in the microbial load, attributed to the pronounced antimicrobial effect exerted by *Aronia* extract. An inhibitory effect in the first week was determined for *Enterobacteriaceae,* which also showed a decrease of 1 log UFC/mL after two weeks of administration. An important observation was the simultaneous increase in the number of strains from the genera *Lactobacillus* and *Bacteroides*, which determined a positive response for the modulation process with an average increase of approximately 0.2 log UFC/mL.

### 3.4. The Effect of Aronia Extract on the Synthesis of Organic Acids

Organic acids (Table 1 and Table S1) showed variations, some of which could be correlated with the evolution of the simulated microbiota fingerprint. The increase in the number of favorable strains in the first month was correlated with the accumulation of up to 0.38 ± 0.004 mg/mL of propionic acid. Butyric acid showed an evolution between 0.6–0.8 mg/mL. This was not directly correlated with the variation in the number of microorganisms in the microbiota pattern or with the ratio of the strains that were determined (Figure 4).

Other organic acids that can be categorized as biomarkers and whose presence can be interpreted as a result of chokeberry extract administration (isovaleric acid) were also determined in small amounts. Lactic acid and acetic acid showed the highest values, having at least double amounts compared to butyric acid, considered to be one of the most important biomarkers. They were not influenced by the prolonged administration of Aronia extract as a modulator of the microbial pattern. The same situation was registered for butyric and benzoic acid. The rest of the acids showed reductions with the extension over 2–3 months of administration of the extract in vitro.

## 4. Discussion

The effects of Aronia extract administration for the control of dysbiotic microbiota were highlighted together with a series of other data that demonstrated a high biological value of the extract from this fruit that can be used in the stages of prevention of some chronic pathologies (diabetes) or as an adjuvant in classic therapies. Microbiota patterning for target groups is a current trend in functional food/product studies [17]. They aim to increase the effectiveness of therapies and decrease the amount of drugs used [18]. The amount of product administered is of essential importance (Figure 4), indicating that the modulatory and cytoprotective effects are valid with regular administration/consumption for at least 2–3 months.

The negative point was that, although the total polyphenolic level was relatively constant (Figure 2), after the third month of the administration, the antioxidant potential decreased due to the action of the microbiota. The data are supported by the identification in the samples of some individual polyphenolic compounds that are included in the composition of Aronia juice. These data observed for the first time showed that the period of administration was critical because the polyphenolic load in Aronia had a strong antimicrobial effect that did not differentiate between bacterial strains. These results were extremely important to understand the microbiota pattern’s response and to modulate the effect that long periods of administration/consumption can have on various functions of the human body [19]. Compared to a previous study [20], the so-called lack of toxicity was contradicted by the long-term administration’s pronounced antimicrobial effect. The polyphenolic pattern had an essential role here, but the in vitro data partially contradicted previous studies conferring a major role in cytoprotection. In the long term, we believe that the non-selective elimination of a part of the microbiota cannot be a positive factor. These data can be supported by the decrease in antioxidant protection, which can be correlated with a decrease in the cytoprotection effect [14].

The identification of polyphenolic compounds in the culture samples during the entire period of the experiments can explain some of the effects of Aronia juice administration. For example, chlorogenic acid, a powerful antioxidant in large quantities in Aronia juice, can prevent glucose metabolic disorders and inhibit metabolic endotoxemia [21]. On the other hand, epicatechin and its main metabolite 5-(3′,4′-dihydroxy phenyl)-γ-valerolactone (3,4-diHPV), can differently influence the activation of the nuclear factor erythroid 2–related factor 2 (Nrf2) and therefore physiological and pathophysiological outcomes of oxidant exposure [22]. Ferulic acid has anti-inflammatory activities and protects intestinal epithelial cells [23]. The identification of coumaric acid shows that in the in vitro tests carried out, there was a metabolism of the anthocyanins from the Aronia extract; it is known that following the metabolism of the anthocyanins, gallic, syringic, and p-coumaric acids are formed [24]. Another identified flavonoid, apigenin, influences the Firmicutes to Bacteroidetes ratio, promotes the synthesis of short-chain fatty acids, and inhibits the population of Enterococcus caccae by altering the gene expression of this microorganism [23]. Modulating and protective effects have also been reported for kaempherol, naringenin, caffeic acid, coumaric acid, ellagic acid, isoquercitin, or rutin [25,26,27,28,29,30,31].

An effect observed for using Aronia extract as a functional supplement would be limiting microbiota and reducing metabolic activity [32,33]. There was no proliferation of dysbiosis, but after a rapid correction (about 1 month of administration), the result was microbiological and metabolomic restriction. The preservation of the synthesis of some essential organic acids (propionic acid, for example—Table 1) was an important result, but the level recorded was quantitatively limited [34]. The administration of this functional product should not be done for a period longer than two, a maximum of three, months to preserve the general effect of microbial and metabolomic modulation. The in vitro effect obtained will be able to be used when it is recommended for specific target groups of the population (children with a predisposition to diabetes or people suffering from type 2 diabetes).

These results will be able to be used in classical therapy and are useful to be able to obtain a therapeutic effect that supports a good state of health. The modulation of the functions of the human microbiota represented a valuable result because the extract limits the proliferation of *E. coli* strains and represents an adjuvant in treating prediabetic conditions or type 2 diabetes [34]. The improvement (modulation) of the possible side effect of a decrease in pancreatic functions can be obtained by a controlled administration of this extract. Special care must be taken when administering the extract to children, where the functioning of the microbiota is essential in maintaining homeostasis due to the limited plasticity of a newly formed pattern [35]. In the long term, Aronia extract can produce changes at the level of the main bacterial groups in the human colon. The most significant changes appear in the first month after administration, but for most groups, they stabilize. After four months of the administration, a ten-fold increase was observed in the case of members of the *Actinomyces* group. Thus, we can say that a long-term administration of the extract may not be recommended due to possible changes in the groups of microorganisms in the microbiota. It would be preferable for the extract to be administered over a course of two months.

## Figures and Tables

**Table 1:** The amount of organic acids obtained after administration for six months of Aronia extract in vitro.

Compound	Blank mgmL^−1^	Week1 mgmL^−1^	Week2 mgmL^−1^	Week3 mgmL^−1^	Week4 mgmL^−1^	Month2 mgmL^−1^	Month3 mgmL^−1^	Month4 mgmL^−1^	Month5 mgmL^−1^	Month6 mgmL^−1^
Formic acid	0.170 ± 0.021	147.0 ± 13.0	0.09 ± 0.012	0.07 ± 0.009	0.068 ± 0.004	0.043 ± 0.004	0.063 ± 0.004	0.063 ± 0.004	0.056 ± 0.002	0.055 ± 0.004
Oxalic acid	0.057 ± 0.004	0.048 ± 0.001	0.030 ± 0.004	0.024 ± 0.004	0.022 ± 0.001	0.015 ± 0.001	0.023 ± 0.003	0.017 ± 0.001	0.020 ± 0.004	0.023 ± 0.001
Succinic acid	0.173 ± 0.04	0.128 ± 0.02	0.083 ± 0.01	0.099 ± 0.02	0.097 ± 0.09	0.061 ± 0.01	0.118 ± 0.03	0.102 ± 0.003	0.11 ± 0.01	0.077 ± 0.07
Acetic acid	2.67 ± 0.02	2.21 ± 0.015	1.77 ± 0.012	1.77 ± 0.043	2.04 ± 0.04	1.72 ± 0.03	3.63 ± 0.05	2.96 ± 0.09	2.92 ± 0.07	2.66 ± 0.06
Propionic acid	0.027 ± 0.002	0.037 ± 0.006	0.035 ± 0.001	0.022 ± 0.001	0.038 ± 0.004	0.027 ± 0.003	0.044 ± 0.005	0.040 ± 0.005	0.027 ± 0.002	0.023 ± 0.001
Lactic acid	0.974 ± 0.156	1.297 ± 0.034	1.145 ± 0.06	1.10 ± 0.072	1.408 ± 0.265	1.508 ± 0.037	1.154 ± 0.077	1.263 ± 0.115	1.274 ± 0.023	1.434 ± 0.061
Butyric acid	0.651 ± 0.034	0.701 ± 0.02	0.608 ± 0.02	0.571 ± 0.034	0.599 ± 0.021	0.633 ± 0.04	0.849 ± 0.046	0.696 ± 0.024	0.687 ± 0.026	0.707 ± 0.021
Isovaleric acid	0.048 ± 0.005	0.042 ± 0.009	0.033 ± 0.005	0.038 ± 0.008	0.055 ± 0.019	0.026 ± 0.004	0.06 ± 0.005	0.04 ± 0.002	0.035 ± 0.001	0.033 ± 0.004
Benzoic acid	0.038 ± 0.001	0.049 ± 0.002	0.056 ± 0.001	0.057 ± 0.001	0.057 ± 0.003	0.05 ± 0.00	0.086 ± 0.002	0.067 ± 0.001	0.072 ± 0.001	0.073 ± 0.00
Phenyllactic acid	0.012 ± 0.002	0.008 ± 0.00	0.006 ± 0.00	0.006 ± 0.00	0.006 ± 0.00	0.006 ± 0.00	0.013 ± 0.002	0.011 ± 0.00	0.012 ± 0.00	0.008 ± 0.00
Hydroxyphenyl lactic acid	0.003 ± 0.00	0.005 ± 0.00	0.004 ± 0.00	0.005 ± 0.00	0.004 ± 0.00	0.002 ± 0.00	0.013 ± 0.00	0.010 ± 0.00	0.009 ± 0.00	0.007 ± 0.00

## Data Availability

Not applicable.

## Supplementary Materials

The following supporting information can be downloaded at https://www.mdpi.com/article/10.3390/life13010032/s1. Figure S1: electropherograms of organic acid separation by zonal capillary electrophoresis; up: standards (in order of elution, formic acid, oxalic acid, succinic acid, acetic acid, propionic acid, lactic acid, butyric acid, isovaleric acid, benzoic acid, phenyllactic acid, and hydroxyphenyllactic acid); down: culture sample after two months of incubation with Aronia extract. Figure S2: chromatogram of polyphenols separation from bacterial culture sample after one week of Aronia extract administration at 280 nm. Figure S3: chromatogram of polyphenols separation from bacterian culture sample after one week of Aronia extract administration at 320 nm. Figure S4: chromatogram of polyphenols separation from bacterial culture sample after one week of Aronia extract administration at 360 nm.
