# Supplementary material for: Dysbiotic Gut Microbiota Modulation by Aronia Fruits Extract Administration
Source: Life (Basel). 2022 Dec 22;13(1):32. doi: 10.3390/life13010032 (PMC9865120; doi:10.3390/life13010032)
Supplement: Supplementary file 1 [file life-13-00032-s001.zip › life-2086714-supplementary.pdf]

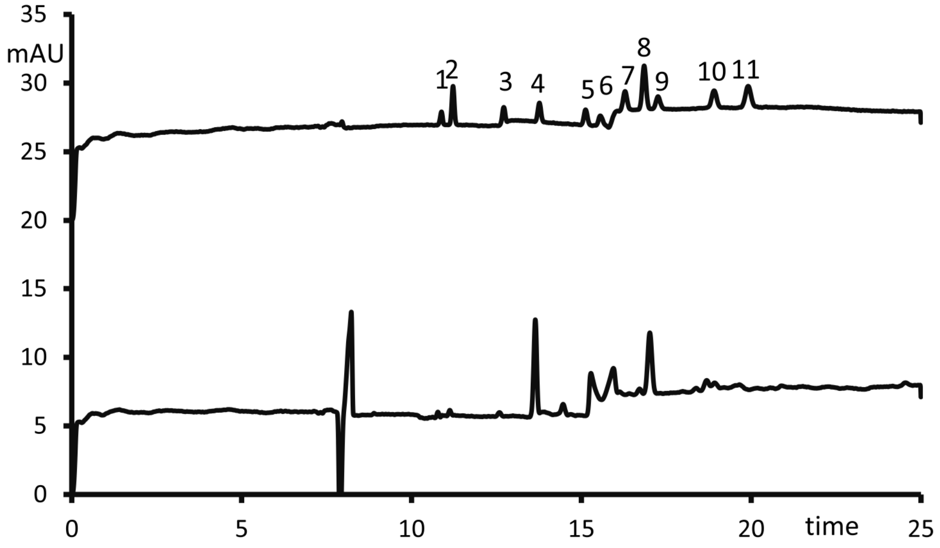

Figure S1. Electropherograms of organic acids separation by zonal capillary electrophoresis; up: standards (in order of elution, 1-formic acid, 2-oxalic acid, 3-succinic acid, 4-acetic acid, 5-propionic acid, 6-lactic acid, 7-butyric acid, 8-benzoic acid, 9-isovaleric acid, 10-phenyllactic acid, 11-hydroxyphenyllactic acid); down: culture sample after 2 month of incubation with Aronia extract

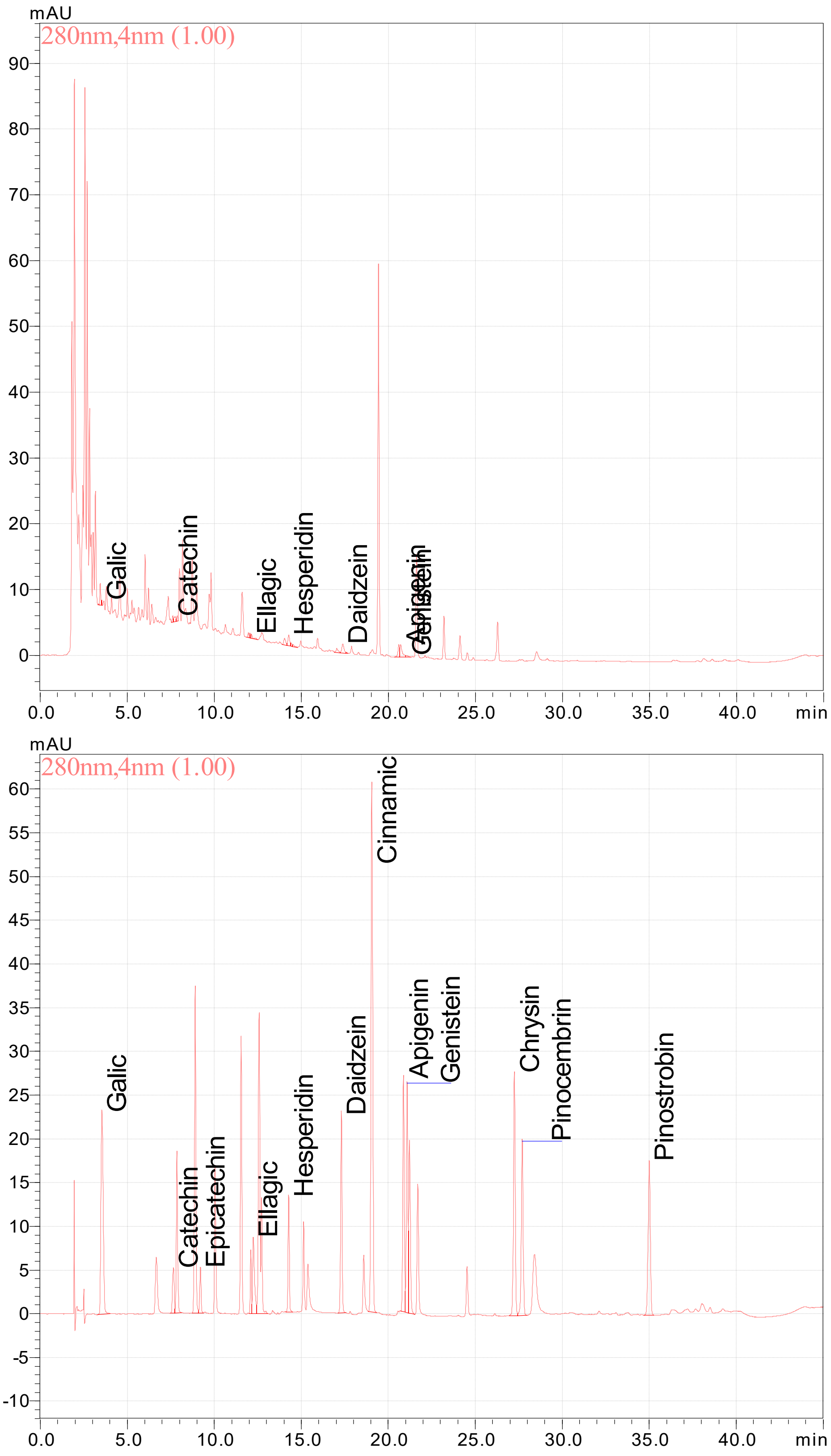

Figure S2. Chromatogram of polyphenols separation from bacterial culture sample after 1 week of Aronia extract administration and chromatogram of standards at 280 nm

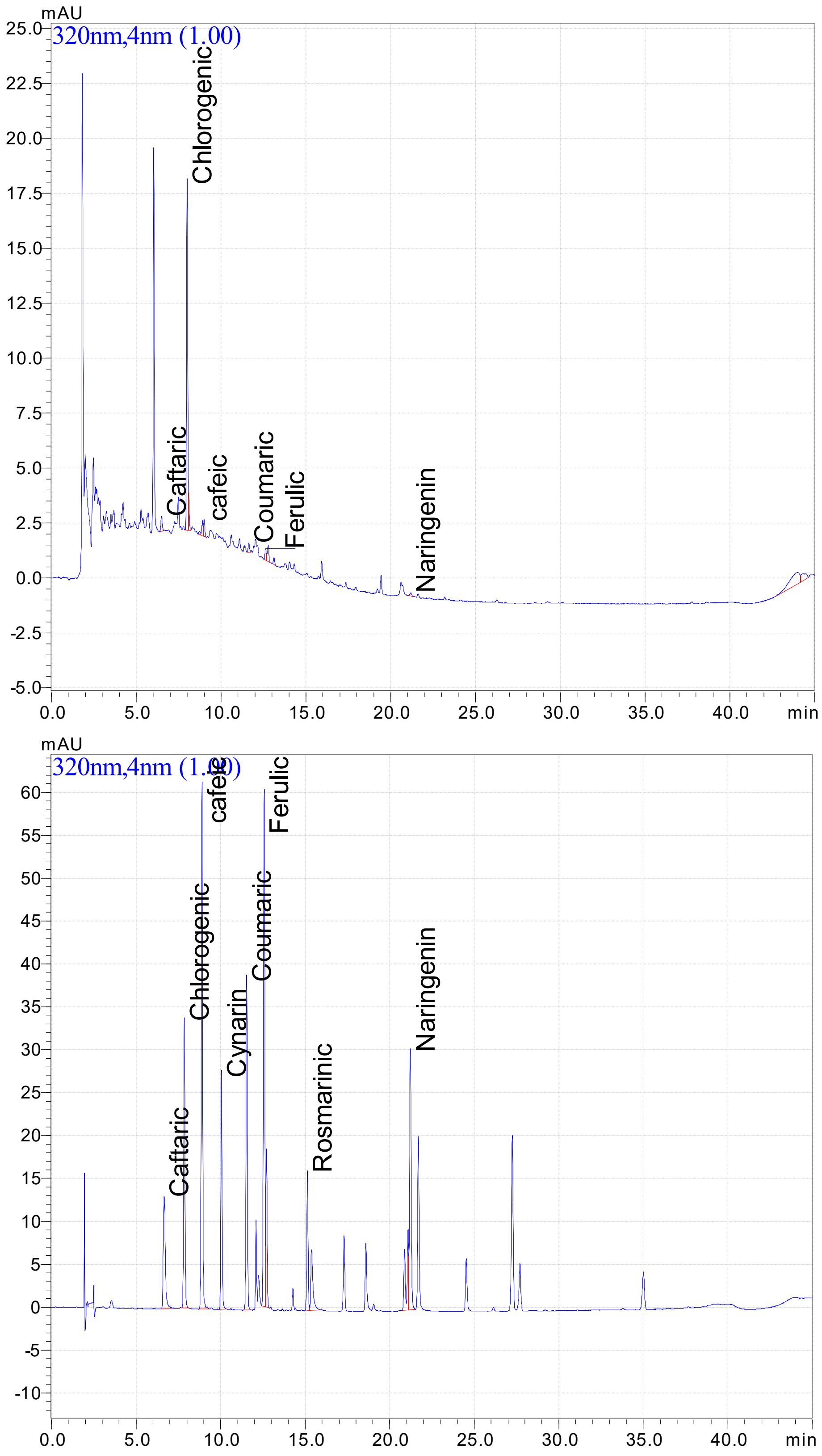

Figure S3. Chromatogram of polyphenols separation from bacterial culture sample after 1 week of Aronia extract administration and chromatogram of standards at 320 nm

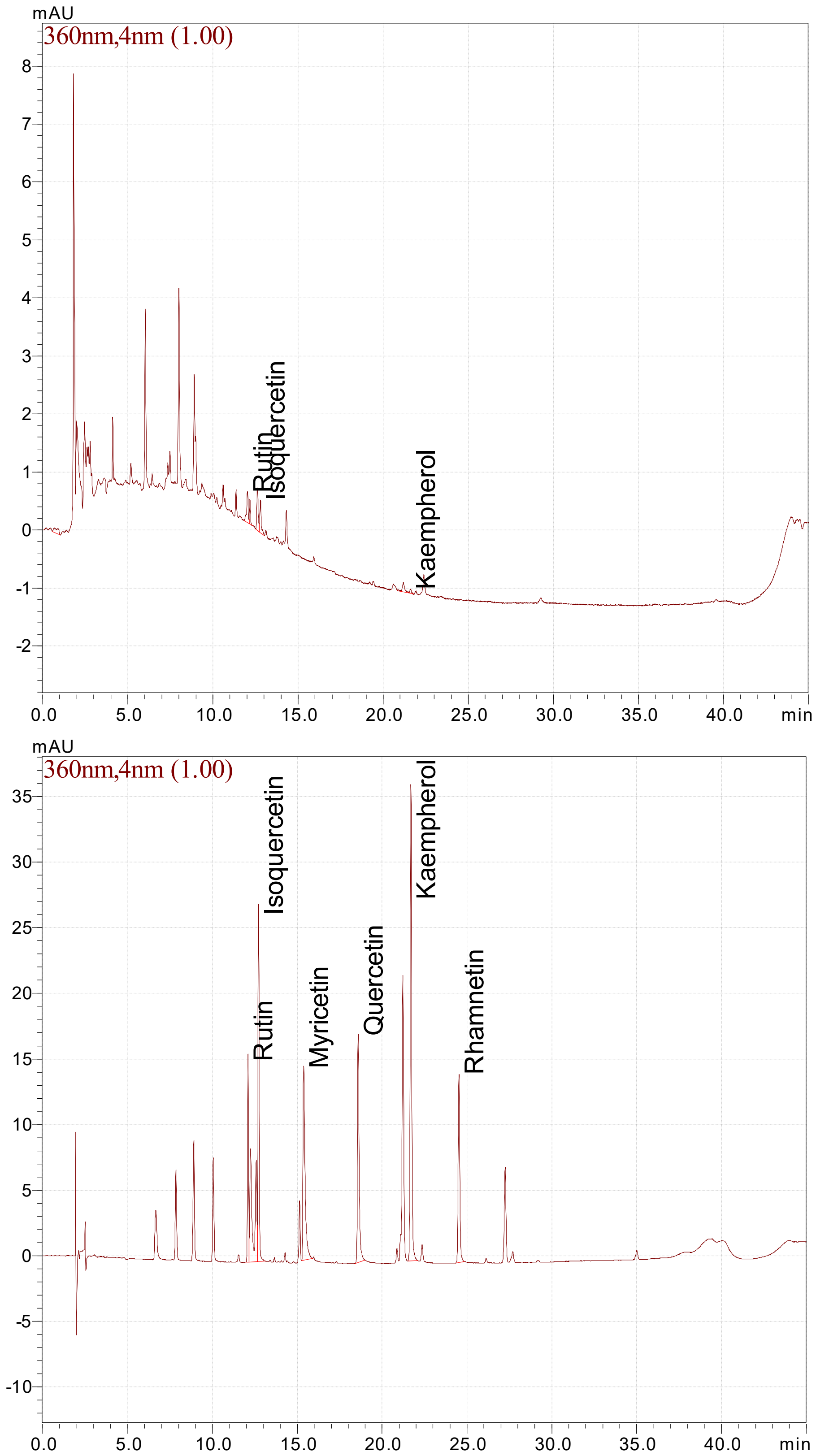

Figure S4. Chromatogram of polyphenols separation from bacterial culture sample after 1 week of Aronia extract administration and chromatogram of standards at 360 nm
